# Supertransient Chaos in a Single and Coupled Liénard Systems

**DOI:** 10.3390/e26100812

**Published:** 2024-09-24

**Authors:** S. Leo Kingston, Suresh Kumarasamy, Agnieszka Chudzik, Jerzy Wojewoda, Tomasz Kapitaniak

**Affiliations:** 1 Division of Dynamics, Lodz University of Technology, Stefanowskiego 1/15, 90-924 Lodz, Poland; agnieszka.chudzik@p.lodz.pl (A.C.); jerzy.wojewoda@p.lodz.pl (J.W.); tomasz.kapitaniak@p.lodz.pl (T.K.); 2 Center for Artificial Intelligence, Easwari Engineering College, Chennai 600089, Tamilnadu, India; sureshscience@gmail.com

**Keywords:** supertransient chaos, Liénard system, Lyapunov exponent, hyperchaos, electronic circuit

## Abstract

We report the appearance of supertransient chaos in a single and two-coupled Liénard system with the influence of external periodic force. The existence of transient dynamics in a model is significantly long before it settles into the asymptotic steady state of periodic dynamics understood as supertransient chaos. The two diffusively coupled forced Liénard systems exhibit extremely long transient dynamics when their frequencies of the external forcing are slightly mismatched. Additionally, the coupled system signifies supertransient hyperchaotic dynamics for a specific set of system parameters. This study involves different numerical characterizations, statistical analysis, and hardware implementation using an analog electronic circuit.

## 1. Introduction

The occurrence of chaotic motion in a finite time interval then switches into regular dynamics known as transient chaos was identified a long time ago. The studies on chaotic and transient chaotic dynamics are related to the temporal evolution of the system with its asymptotic dynamics. For modeling, predicting, and controlling processes, these transient dynamics are more important than the system’s steady state [1,2]. Hence, understanding the emerging process of transient chaos is more important in different disciplines based on the perspectives of the nonlinear dynamical system. This phenomenon was explored in conservative and dissipative systems of mathematical models, maps, and also some of the laboratory-based experiments [1]. Tél has recently explained various applications of transient dynamics, including insight into finding the decision-making solutions and comprehending the dynamics of aerosol particles that aid in controlling air pollution [3]. A coupled pendulum model with numerical simulations was used to show the formation of transient chaotic synchronization dynamics. This was confirmed by an experiment performed in real time [4]. Moreover, the complete comprehension of the brain activities, memory effects in particle dispersion, and intuition of the nature of turbulence have been explored based on transient dynamics [3,5].

On the other hand, only a few models have shown that there is transient dynamics over a very long time period. This is called supertransient chaos (STC) [1,6,7,8,9]. A one-dimensional metapopulation model exhibits STC which are rare and highly influenced by the choice of initial conditions [6]. In general, the time scale of the ecological interest is ten or hundred, whereas some specific ecological models persist supertransient dynamics of thousand generations or even more have been reported in the literature; for more details, see refs. [7,8]. Additionally, the formation of STC has been reported in a coupled lattice model, in which the average lifetime of transient dynamics is very large owing to the impact of chaotic saddle [9].

In this present study, to elucidate the existence of supertransient chaotic dynamics, we have taken the driven Liénard system, which is one of the paradigmatic models of nonlinear dynamical systems. This model has been extensively studied in recent years to disclose distinct intricate dynamics [10,11,12,13]. First, we demonstrate the emergence of supertransient chaotic dynamics in a single forced Linéard model for a specific set of system parameter values. Further, to illustrate the generality of our observation, we explore the STC dynamics in a two diffusively coupled Linéard system. In addition, the coupled Liénard manifests supertransient hyperchaotic motion inclusive of STC dynamics.

The existence of hyperchaos in the higher dimensional systems is described as the appearance of two directional stretching and folding of the systems’ trajectories, resulting in the occurrence of more complex dynamics compared with the chaotic motion. The emergence of hyperchaotic systems is widely reported in maps [14], continuous time dynamical systems [15,16], and delay models [17,18]. We have observed that the coupled Liénard system proves supertransient hyperchaotic dynamics for a specific choice of parameter value.

The remaining part of this work is organized as follows; the detailed description of the externally excited Liénard system and its parameters discussed in Section 2. The formation of supertransient chaotic and hyperchaotic dynamics in single and coupled Liénard systems using various dynamical and statistical analyses elaborated in Section 3 and Section 4, respectively. The numerical observation of supertransient chaotic dynamics was validated using an analog electronic experiment presented in Section 5. The comprehensive overview of our observation is illustrated in Section 6.

## 2. Externally Excited Liénard System

In this study, we considered a specific class of Liénard equation of the following form:

(1) $$\begin{array}{c} \ddot{x}+\alpha x\dot{x}+\gamma x+\beta x^{3}=0 \end{array}$$

where α, β, and γ illustrate the position-influenced damping coefficient, nonlinearity strength, and natural frequency of the system, respectively. $\ddot{x}+f\left(x\right)\dot{x}+g\left(x\right)$ = 0 was the generic form of Equation (1) satisfying the Liénard equation. f(x)=αx and $g\left(x\right)=\gamma x+\beta x^{3}$ are the variables under consideration. Depending on the magnitude of parameter values γ and β, we have identified four different physical situations such as single well, double well, single hump, and double hump as similar to the standard Duffing oscillator. In the present, we have used a double well physical situation, i.e., β> 0 and γ< 0. In addition to calculating the system stability [19], Equation (1) is written as follows:

(2) $$\begin{array}{c} y=0\\ -\alpha xy-\beta x^{3}-\gamma x=0. \end{array}$$

From Equation (2), we have obtained the fixed points of the system as (0, 0), ($\pm \frac{\gamma }{\beta },0$). When we select the parameters value β = 0.5 and γ = (−0.5, the autonomous Liénard system exhibits distinct stability such as the saddle at the origin, the unstable focus at (−1, 0), and the stable focus at (1, 0), respectively.

To identify the different complex dynamics in the considered model, we have applied the external sinusoidal voltage source Asin(ωt) in Equation (1). Hence, the externally excited Liénard system read as the following form of differential equations;

(3) $$\begin{array}{ccc} \dot{x} & = & y\\ \dot{y} & = & -\alpha xy-\beta x^{3}-\gamma x+A\sin\left(\omega t\right). \end{array}$$

Here, *A* and ω are the amplitude and frequency of the external sinusoidal forcing term, respectively. While applying external periodic forcing, the Liénard system exhibits different complex dynamics. For example, various configurations of model Equation (3) shows a large variety of intricate dynamics, such as bursting and spiking dynamics [10,11], successive periodic and chaotic mixed-mode oscillations [20], extreme events [12,13], and others [21], have been reported in the literature. Here, we illustrate the emergence of supertransient chaos and supertransient hyperchaotic dynamics for a precise choice of system parameters that will be elaborated on in more detail in the upcoming sections.

## 3. Supertransient Chaos: Single Liénard System

**Figure 1:**
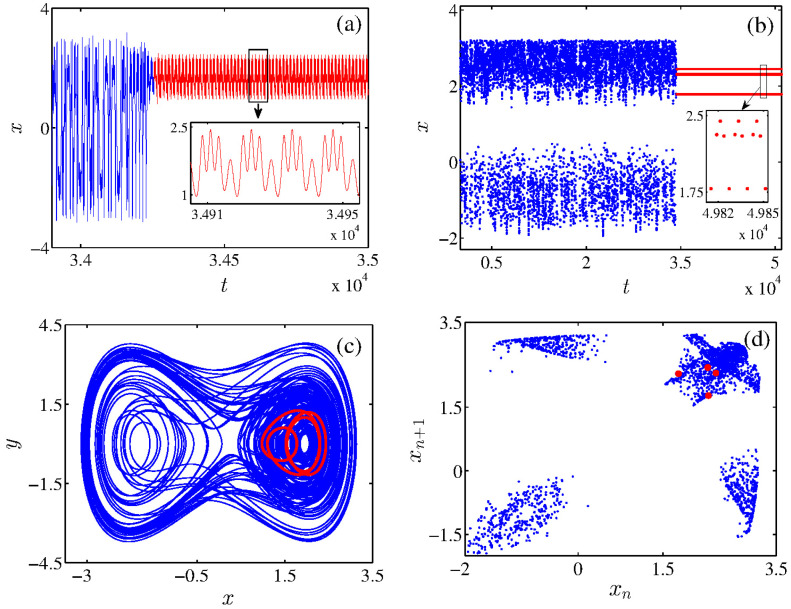
Numerical results of supertransient chaos: (**a**) time series exhibits transient dynamics (blue) of the system state variable x(t) up to t≈ 34,220, then it settles into the asymptotic steady state of periodic motion (red), it insert figure shows the magnified version of the steady state of period four attractor. (**b**) Peaks of time series for long run data to probe the relatively long transient state, then transit to the steady state motion. (**c**) Phase portrait of transient chaos in the (x−y) plane and (**d**) Poincaré return map in the ($x_{n}-x_{n+1}$) plane.

To demonstrate the existence of supertransient chaos in a single Liénard system, we have solved Equation (3) utilizing 4th order Runge–Kutta integration method. Note that we choose the initial conditions at closer to zero for all numerical simulations. The parameters value of the system fixed as α = 0.0135, β = 0.8111, γ = 2.65, *A* = 1.5, and ω = 0.52. We have obtained STC dynamics for this precise choice of system parameters. We have presented the temporal dynamics of STC for adequate range, and also the peaks of time series for the long run of the data set, as shown in Figure 1a,b. The existence of the very long transient state (blue) of the system before it settles into the asymptotic steady state (red) of the periodic attractor is well obtained from the time series plots (see Figure 1a,b). Indeed, the model proves an extremely long transient time compared with the usual transient dynamics reported in the literature. On account of that, we described this dynamic as supertransient chaos. The insert plots in Figure 1a,b signify the magnified version of regular dynamics (period-four attractor).

For better visualization of the dynamics, the phase-space of STC is showcased in Figure 1c. The evaluations of chaotic (blue) and regular (red) dynamics are clearly visible in phase portraits of Figure 1c. Broadly, the Poincaré return map analysis is used to discriminate various complex dynamics in a larger class of nonlinear dynamical systems. Here, we have evaluated the Poincaré return map for a system state variable *x* and the obtained results as shown in Figure 1d in the ($x_{n}-x_{n+1}$) plane. It is clearly understood from the return map plot that the large four points (red) reveal the periodic four dynamics owing to the existence of the asymptotic steady state of period four oscillations in the system. However, the randomly distributed points (blue) denote the chaotic oscillations. Next, we have used distinct statistical analysis to characterize further the existence of supertransient dynamics in the forced Liénard system.

### Statistical Analysis

**Figure 2:**
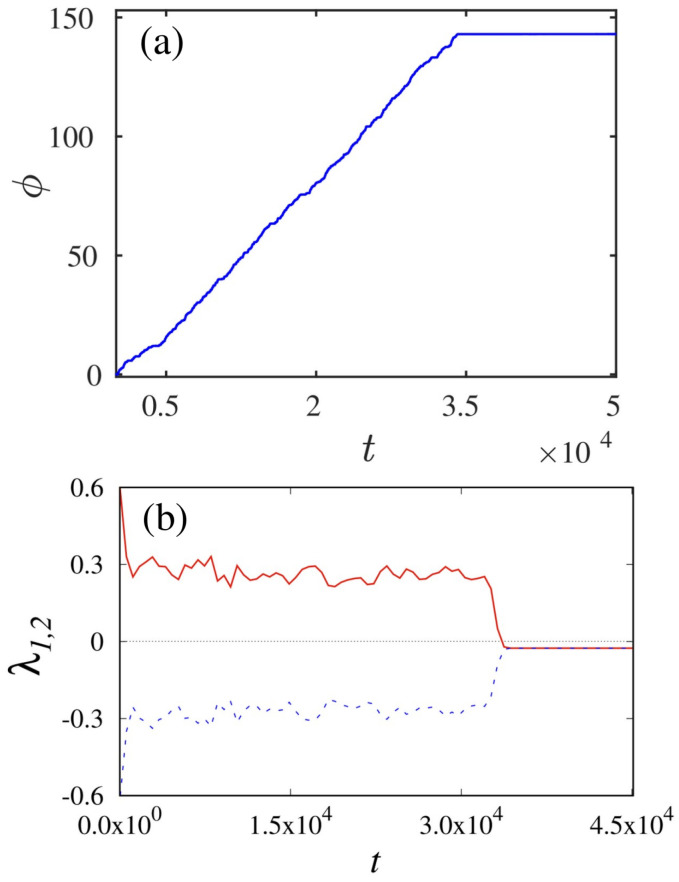
(**a**) Phase (ϕ) measure of a system state variable x(t) which is calculated by employing Hilbert transformation method and (**b**) FTLE spectrum of single Liénard system manifests positive values for the finite time interval (t≈ 34,220) and switched into regular periodic state represented by the negative values of Lyapunov spectrum.

Next, we have estimated the instantaneous phase (ϕ) for the time series x(t) of supertransient chaotic dynamics. To perform the phase analysis, we used the Hilbert transformation method [22]. The phase measure (ϕ) of state variable x(t) in the (t−ϕ) plane is portrayed in Figure 2a. The phase plot (ϕ) of Figure 2a reveals that the phase values of the system monotonically increased up to the transient dynamics. After that, it attains a constant value for the periodic dynamics. As a consequence, the advent of both chaotic and asymptotic steady states in supertransient chaos are validated using phase analysis. In addition, we characterize the existence of STC dynamics using the reliable statistical analysis of the finite-time Lyapunov exponent (FTLE) method. The procedure for calculating the Lyapunov exponent values is given in ref. [23]. The calculated FTLE values unveil the information about the amount of stretching (or folding) of trajectory for the finite time interval. The FTLE values for the suitable range of time intervals are depicted in Figure 2b. Precisely, the FTLE plot of Figure 2b signifies that the first Lyapunov exponent (solid red line) resides in the positive values for a relatively long time of transient state. On the other hand, the negative values of the exponent represent the regular periodic motion. Extensively, we have used distinct statistical analyses to demonstrate the emergence of the STC phenomenon in the forced Liénard system.

## 4. Supertransient Chaos: Coupled Liénard Systems

Further, to elaborate on the commonality of our observation, we have examined the appearance of supertransient chaos in a two-coupled Liénard system. Specifically, we have considered diffusively coupled configuration, and its corresponding equations are expressed as the four coupled first-order equations below:

(4) $$\begin{array}{ccc} \dot{x_{1}} & = & y_{1}\\ \dot{y_{1}} & = & -\alpha x_{1}y_{1}-\beta x_{1}^{3}-\gamma x_{1}+A_{1}\sin\left(\omega_{1}t\right)+k\left(x_{2}-x_{1}\right)\\ \dot{x_{2}} & = & y_{2}\\ \dot{y_{2}} & = & -\alpha x_{2}y_{2}-\beta x_{2}^{3}-\gamma x_{2}+A_{2}\sin\left(\omega_{2}t\right)+k\left(x_{1}-x_{2}\right) \end{array}$$

**Figure 3:**
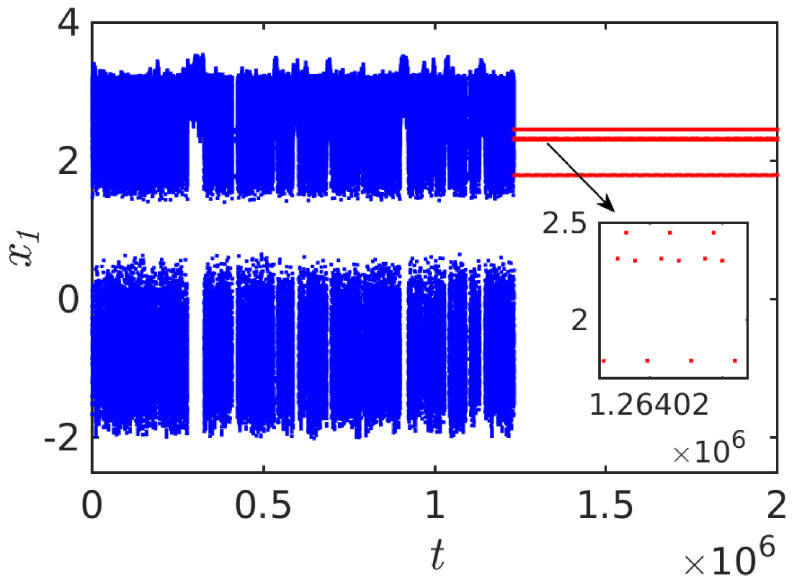
Time series of $x_{1}\left(t\right)$ (local maxima for a long time interval) for diffusively coupled Liénard system exhibits extreme transient dynamics (blue) and it settles into the invariant stable state of periodic motion (red). The magnified region of the periodic state is presented as an insert plot.

In Equation (4), α, β, γ, and *k* are the nonlinear damping, strength of nonlinearity, internal frequency, and coupling coefficient, respectively. Additionally, $f_{1}$, $f_{2}$, $\omega_{1}$, and $\omega_{2}$ are the amplitudes and frequencies of the external sinusoidal forces of the system. The system parameters are set as α = 0.0135, β = 0.8111, γ = −2.65, and $f_{1}$ = $f_{1}$ = 1.5. Also, we have chosen a small mismatch in the external forcing frequencies as $\omega_{1}$ = 0.52 and $\omega_{2}$ = 0.5198. For this specific parametric choice, we identified STC in the diffusively coupled Liénard system. The temporal evolution of STC is shown in Figure 3. In Figure 3, we have presented the local maxima (peaks) of the time series for clear visualization. The system exhibits transient motion $t\approx 1.2334\times 10^{6}$ (blue points), and then switches into the regular dynamics (red points) evident in the time series. The insert plot of Figure 3 manifests an asymptotic steady state of period four oscillations. The coupled system exhibits a significantly longer transient lifetime due to the effect of mismatch parameters in the system.

### Supertransient Hyperchaos

**Figure 4:**
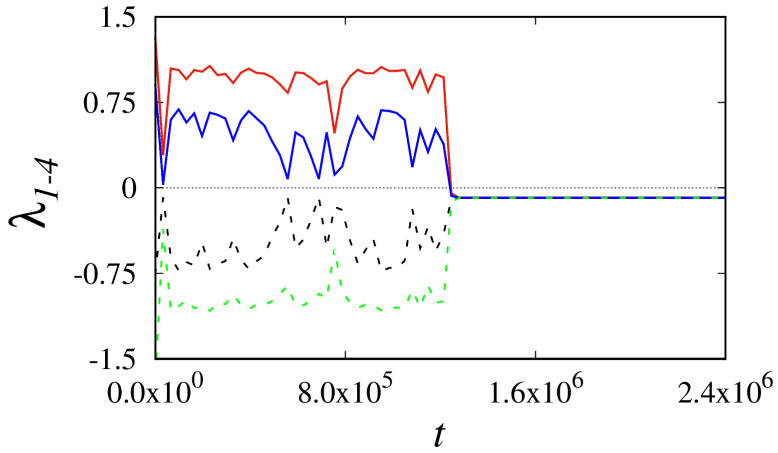
FTLE spectrum of system Equation (4) for the finite time interval, positive values of the spectrum signify transient dynamics and regular periodic state represented by the negative values of Lyapunov spectrum.

Further, to confirm the advent of supertransient chaos in the two-coupled-driven Liénard systems, we have estimated the finite time Lyapunov exponent values for Equation (4). The obtained FTLE spectra are plotted for the finite time interval presented in Figure 4. Intriguingly, we have identified the first two positive largest Lyapunov exponents (solid red and blue lines) for the chaotic domain, followed by its transit into the negative values for the regular dynamics. The existence of two positive Lyapunov exponents reveals the emergence of hyperchaotic dynamics. Certainly, hyperchaotic dynamics can be obtained only in the higher-dimensional system of at least the fourth-order model in the continuous time dynamical systems. Here, the coupled Liénard system proves supertransient hyperchaos for an appropriate choice of system parameters. The other two Lyapunov exponent values are represented as dashed black and green lines in Figure 4. Overall, we have used different numerical analyses of dynamic and statistical measures to confirm the existence of supertransient dynamics in isolated and diffusively coupled Liénard systems. In the following section, we have validated our observation using a real-time hardware electronic experiment.

## 5. Experimental Results

**Figure 5:**
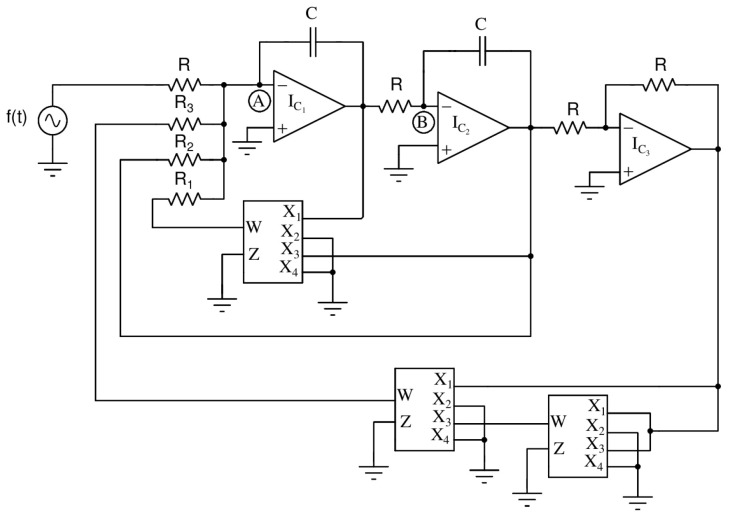
Analog circuit for forced Liénard system. The circuit output is measured at two nodes of the capacitors *A* and *B*. Also, f(t) denotes the external sinusoidal function generator. The circuit parameter values are explained in the text.

We observed the supertransient chaos experimentally by using an electronic experiment. For that, we have designed an analog electronic circuit for Equation (3). The analog circuit schematic for our proposed model is depicted in Figure 5. The circuit is set up in the laboratory using analog device multipliers (AD633 JN), operational amplifiers (TL082), fixed capacitors, variable and constant resistors, and external sinusoidal forcing sources [24]. The output of the circuit at junctions *A* and *B* can be written as Equations (5).

(5) $$\begin{array}{ccc} U & = & -\frac{1}{C}\int \left\{-\frac{UV}{R_{3}}+\frac{V}{R_{2}}-\frac{V^{3}}{R_{1}}+\frac{1}{R_{1}}+A\sin\left(\omega t\right)\right\}dt\\ V & = & -\frac{1}{C}\int \left\{\frac{U}{R}\right\}dt \end{array}$$

We differentiate Equations (5) with respect to *t*, and then multiply them by *R*. Followed by rearranging the equations, we have obtained the circuit Equation (6).

(6) $$\begin{array}{ccc} Rc^{2}\frac{d^{2}V}{dt^{2}}-\frac{RC}{10R_{3}}\frac{VdV}{dt}-\frac{V}{R_{2}}+\frac{V^{3}}{100R_{1}} & = & \frac{1}{R}A\sin\left(\omega t\right) \end{array}$$

Moreover, when using the transformation terms of t/RC=t, U=x, V=y and $\omega =RC\omega^{\prime }$ in Equation (6) for the resealing view point, after dropping the prime in the *t*, the normalized equation for theoretical convenience is represented as Equation (7).

(7) $$\begin{array}{ccc} \ddot{x}-\frac{R}{10R_{3}}x\dot{x}-\frac{R}{R_{2}}x-\frac{R}{100R_{1}}x^{3} & = & A\sin(\omega^{\prime }t) \end{array}$$

**Figure 6:**
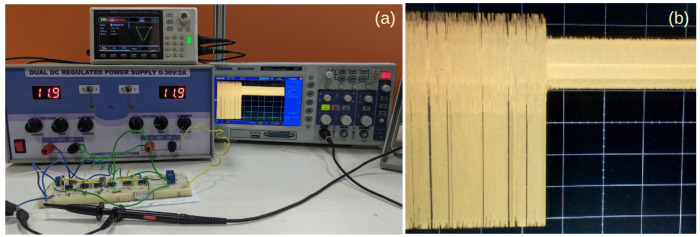
(**a**) Experimental setup of forced Liénard system and (**b**) the snap short of time series $v_{1}\left(t\right)$ shows the long transient dynamics and followed by regular period-four attractor.

We have fixed R = 10 KΩ, C = 10 nF, and the remaining circuit parameters values are chosen as R1 = 271 KΩ, R2 = 28.36 KΩ, R3 = 248 KΩ. Also, the external forcing parameters are fixed as *A* = 1.5 V and ω = 1.1 KHz. The circuit exhibits supertransient chaos dynamics for these precise parameter values. The complete experimental setup of the forced Liénard system, we constructed in the laboratory is showcased in Figure 6a. The snapshot of the experimental time series observed at node *A* is presented in Figure 6b. The experimental time series proves the appearance of a long transient time before it settles into a steady state. Furthermore, we have observed that the experiment result agrees with the numerical simulation results.

## 6. Conclusions

We have presented the emergence of supertransient chaos in both single and coupled-driven Liénard for the suitable choice of the system parameters. The appearance of transient dynamics is significantly longer in two diffusively coupled systems when incorporating the small mismatch in their forcing frequency values. In addition, we have identified supertransient hyperchaos in a coupled system verified by the presence of two positive Lyapunov exponents. The formation of the STC phenomenon was confirmed by using different dynamical analyses of time evaluations, phase portraits, Poincaré return map, and phase analysis using Hilbert transformation. Moreover, the statistical measure of the finite-time Lyapunov exponent shows the existence of both chaotic and periodic dynamics in a finite time interval. Finally, we have validated our numerical investigations with experimental results using real-time hardware electronic circuit. The obtained numerical and experimental results are well aligned with each other.

## Data Availability

The original contributions presented in the study are included in the article, further inquiries can be directed to the corresponding author.
